# Supplementary figures and images for: Aβ Aggregates Bind the U1 Spliceosomal Ribonucleoprotein in Alzheimer Disease Brain
Source: bioRxiv. 2026 Jun 5:2026.06.02.729610. Preprint. [Version 1] doi: 10.64898/2026.06.02.729610 (PMC13252199; doi:10.64898/2026.06.02.729610)

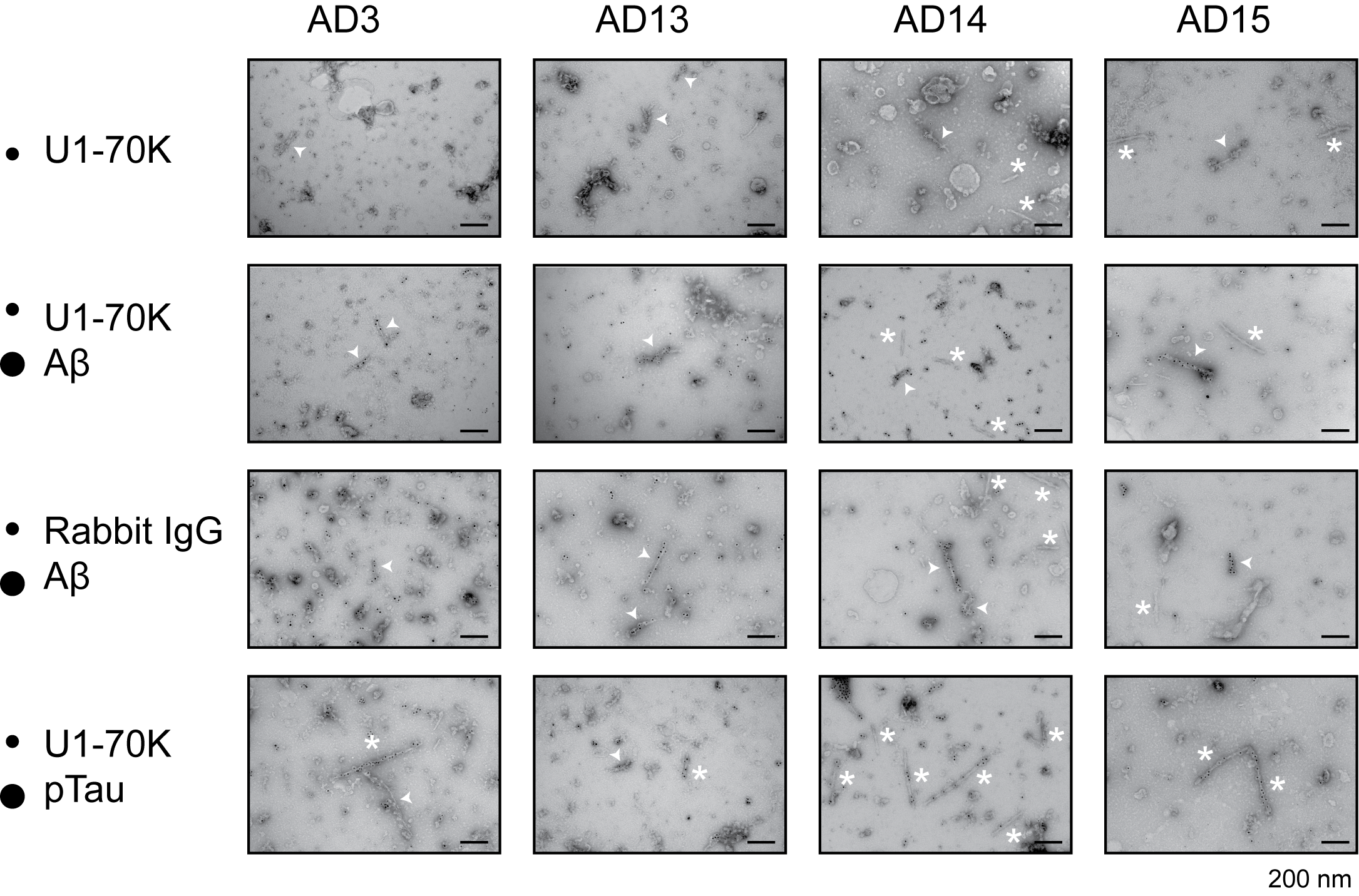

Supplement: Supplement 3 [file media-3.tif]
